# Effect of calcium glucoheptonate on proliferation and osteogenesis of osteoblast-like cells in vitro

**DOI:** 10.1371/journal.pone.0222240

**Published:** 2019-09-09

**Authors:** Prashant Kumar Modi, Ashwini Prabhu, Yashodhar P. Bhandary, Sudheer Shenoy P., Aparna Hegde, Sindhu Priya ES, Renjith P. Johnson, Shankar Prasad Das, Sahil Vazirally, Punchappady-Devasya Rekha

**Affiliations:** 1 Yenepoya Research Centre, Yenepoya (Deemed to be University), Mangalore, Karnataka, India; 2 Global Calcium Pvt. Ltd., Bangalore, Karnataka, India; Università degli Studi della Campania, ITALY

## Abstract

Calcium is the key macromineral having a role in skeletal structure and function, muscle contraction, and neurotransmission. Bone remodeling is maintained through a constant balance between calcium resorption and deposition. Calcium deficiency is resolved through calcium supplementation, and among the supplements, water-soluble organic molecules attracted great pharmaceutical interest. Calcium glucoheptonate is a highly water-soluble organic calcium salt having clinical use; however, detailed investigations on its biological effects are limited. We assessed the effects of calcium glucoheptonate on cell viability and proliferation of osteoblast-like MG-63 cells. Calcium uptake and mineralization were evaluated using Alizarin red staining of osteoblast-like MG-63 cells treated with calcium glucoheptonate. Expression of osteogenic markers were monitored by western blotting, immunofluorescence, and qRT-PCR assays. Increased proliferation and calcium uptake were observed in the MG-63 cells treated with calcium glucoheptonate. The treatment also increased the expression of osteopontin and osteogenic genes such as collagen-1, secreted protein acidic and cysteine rich (SPARC), and osteocalcin. Calcium glucoheptonate treatment did not exert any cytotoxicity on colorectal and renal epithelial cells, indicating the safety of the treatment. This is the first report with evidence for its beneficial effect for pharmaceutical use in addressing calcium deficiency conditions.

## Introduction

Calcium is the most abundant mineral in the body with distinct physiological roles such as key regulator of signal transduction pathways of neurotransmission, muscle contraction and fertilization [1]. Extracellular calcium is critical for bone and teeth formation, blood clotting, nerve impulse transmission, regulation of heartbeat and fluid balance within cells as well as in maintaining the action potential across the cell membrane of excitable cells. Bone tissue acts as a reservoir for calcium, in order to balance the concentrations of calcium in muscle, serum and intracellular fluids [2]. The requirements peak during the period of growth such as childhood, during pregnancy and while breast feeding. It is known to promote osteogenesis by amplifying the effect of BMP2 on SMAD signaling [3]. In aging adults, bone resorption exceeds formation, thereby leading to osteoporosis. Also, long term inadequate intake of dietary calcium causes osteopenia or reduced bone density leading to osteoporosis; muscle cramps, premenstrual cramps, insomnia, dermatitis, cardiovascular disease, high blood pressure, blood clotting, and loss of appetite [4, 5]. It can also contribute to alopecia, eczema and psoriasis [6, 7]. Alteration in calcium levels is critical in nervous system disorders such as dementia and depression.

The global calcium map reveals that many countries have low average calcium intake. According to the International Osteoporosis Foundation (https://www.iofbonehealth.org/facts-statistics) [8–10], 1 in 3 women and 1 in 5 men over the age of 50 years experience an osteoporotic fracture. 125 million people are estimated to have osteoporosis in Europe, India, Japan, and the USA alone[11]. Calcium deficiency can be overcome by supplementation with calcium salts. The most common salts used are; calcium carbonate, calcium citrate malate, calcium chloride, calcium gluconate, calcium gluceptate, calcium glubionate, and calcium acetate. These salts differ in the calcium content, solubility, bioavailability and taste[12]. Calcium supplements vary in their absorption rates, depending on their solubility. The low solubility of the inorganic calcium salts such as calcium carbonate is one of the major limiting factors in rapid absorption[13, 14]. To improve the bioavailability of inorganic calcium salts, fabrication of the material into nanostructures have been attempted [15].Although calcium carbonate has the highest calcium content, organic salts are more preferred, due to their water soluble property and enhanced bioavailability and absorption of organic calcium salts.

Calcium glucoheptonate is the water-soluble calcium salt of gluconic acid and in solution, calcium is present both in the free ionized form and as calcium glucoheptonate complex[16, 17]. It is used in the treatment of low blood calcium, high blood potassium, magnesium sulphate overdose, hydrofluoric acid burns, hypocalcemia due to neonatal tetany and intestinal malabsorption, for replenishing the electrolytes (http://www.drugbank.ca/drugs/DB00326).

Calcium has a direct role in osteogenesis by increasing the expression of osteopontin and osteocalcin, thereby promoting osteogenesis [18]. Calcium availability enhances the expression of oestrogenic markers namely, alkaline phosphatase (ALP) and collagen -1. The osteogenic ability of calcium via activation of SMAD and RAS family genes is reported [19].

Compounds other than calcium such as valproic acid and FBS have promoting role in osteogenic differentiation of mesenchymal stem cells (MSCs) leading to bone formation *in vivo* [20, 21] and inhibition of miR-34a has been shown as a key strategy in MSC based therapy by inducing their differentiation [22].

Detailed investigations on the molecular action of calcium glucoheptonate on osteogenesis are lacking. Hence, this study was designed to assess the effect of calcium glucoheptonate with higher calcium equivalents on osteoblast-like cells in vitro using cellular and molecular approaches.

## Materials and methods

### Cell lines and materials

Osteoblast-like cells (MG-63), colorectal epithelial cells (Caco_2_) and kidney epithelial cells (HEK 293T) were procured from National Centre for Cell Sciences (NCCS), Pune, India. Calcium glucoheptonate was kindly supplied by Global Calcium Pvt. Ltd. (Bangalore, India). All the chemicals were of cell culture grade or analytical grade. Calcium glucoheptonate was prepared in sterile milli-Q water to obtain a final concentration of 25 mg calcium per mL. Details of the antibodies and the reagents used in the study are provided in S1 Table.

### Physicochemical characterization of calcium glucoheptonate

The Fourier-transform Infra-red (FT-IR) spectra of calcium glucoheptonate were recorded on a Shimadzu-IRSpirit spectrophotometer at room temperature, and the spectra were taken at the range of 3500−500 cm^−1^. The scanning electron microscopy (SEM) images of the calcium glucoheptonate and EDX analysis were conducted with a Carl Zeiss-FESEM (Zeiss 1540 XB).

### Cell culture conditions

Osteoblast-like MG-63 cells were grown in DMEM media containing 15% Fetal bovine serum (FBS) and antibiotic-antimycotic solution (10000 U Penicillin, 10 mg Streptomycin, and 25 μg amphotericin B). The Caco2 and HEK 293T cells were cultured in DMEM containing 10% FBS and antibiotic-antimycotic solution. The cultures were maintained in T-75 flasks and incubated at 37 ^o^C with 5% CO_2_.

### Cell viability and cell proliferation assays

Effect of calcium glucoheptonate on MG-63, Caco2 and HEK 293T cells were assessed using Methyl Thiazolyl Tetrazolium (MTT) assay [23]. For MTT assay, the cells were seeded at a density of 5000 cells per well in a 96 well microtiter plate and incubated at 37 ^o^C with 5% CO_2_ for 24 hours. Subsequently, the cultures were treated with 25 mg/mL calcium glucoheptonate aqueous solution in calcium equivalents of 0, 0.125, 0.25, 0.5, 1.0, 2.0 and 4.0 mM. After 48 hours, the spent media from each well was removed, and 100 μL of MTT reagent was added. The plate was incubated at 37°C, 5% CO_2_ for 4 hours and the formazan crystals formed were solubilized in 100 μL of DMSO. The viability of the cells was evaluated by reading the absorbance at 570 nm using a Multimode plate reader (FluoSTAR Omega, BMG Labtech). The percentage of cell viability was calculated with reference to the untreated control. Cell viability less than 70% was considered as toxic [24]

The effect of calcium glucoheptonate on the viability of MG-63 cells was further evaluated using trypan blue dye exclusion assay. For this, the cells were seeded at a density of 10,000 cells per well in a 24 well microtiter plate and incubated at 37 ^o^C with 5% CO_2_ for 24 hours. Subsequently, the cells were treated with different concentration of calcium glucoheptonate solutions (0, 0.125, 0.25, 0.5, 1.0, 2.0 and 4.0 mM). After 48 hours of treatment, the cells were trypsinized and resuspended in fresh media. 10 μl of the cell suspension was mixed with 10 μl of trypan blue dye, loaded on to a hemocytometer and counted.

Acridine orange and ethidium bromide (Himedia, India) staining were used for the imaging of the cells [25]. For this, MG-63 cells were seeded (50,000 cells per well) onto 6 well plates and incubated with selected concentrations of calcium glucoheptonate for 48 hours. Cells were washed with PBS, fixed in chilled methanol and stained with acridine orange and ethidium bromide. The stained cells were observed using a fluorescent cell imager (ZOE, BioRad).

### Alizarin Red S staining for calcium uptake

Calcium uptake by MG-63 cells was monitored by incubating cells in 6 well plates with selected concentrations of calcium glucoheptonate for seven days. After the incubation period, the treated cells were washed with PBS and fixed in 4% paraformaldehyde at room temperature for 20 minutes. The fixed cells were stained with freshly prepared alizarin red (2% aqueous, pH 4.2) and washed with PBS before microscopic observation. Images of the stained cells were captured using an inverted microscope (PrimoVert, Carl Zeiss) under 10X magnification. The intensity of the stain uptake was used to assess the calcium accumulation and compared with the untreated control [26]. Alizarin red stain, from the cells, was extracted using 10% acetic acid and quantified at 405 nm using a multimode microplate reader (FluoSTAR Omega, BMG Lantech).

### Alkaline phosphatase activity

Changes in the alkaline phosphatase activity in calcium glucoheptonate treated MG-63 cells were assessed by culturing them for seven days with fresh media supplementation every alternate day. The total alkaline phosphatase activity was measured from culture supernatant and cell lysate. The culture supernatant was used directly for the enzyme activity. The cell pellet was resuspended in cell lysis buffer (Tris-HCl, 50 mM, pH 7.5) and sonicated (30% amplitude, 30 seconds, two cycles). The activity was quantified using the enzyme kinetic method at 37 ^o^C using the commercial kit (Agappe Diagnostics Ltd. India) according to the manufacturer’s instructions [27]. Enzyme activity in the culture supernatant and the cell lysate was recorded using a semi-automatic biochemical analyzer (Mispa Plus, Agappe Diagnostics Ltd. India). Total alkaline phosphatase activity was calculated and expressed as IU/L.

### Western blotting and immunofluorescence

The MG-63 cells (1 x 10^6^) treated with calcium glucoheptonate were harvested by centrifugation (12000 rpm for 30 minutes at 4°C), the protein was extracted using lysis buffer (50 mM Tris-HCl, 0.5% SDS, 250 mM NaCl, 5 mM EDTA, 50 mM NaF) and quantified by BCA method [28]. An equal amount of the protein was resolved on 10% SDS-polyacrylamide gel electrophoresis (SDS-PAGE). After SDS-PAGE, proteins were transferred to PVDF membrane, and immunoblotting was performed. The membrane was blocked with 5% BSA and incubated with primary antibodies for osteopontin, osteocalcin, collagen1, cleaved caspase 3 and cleaved PARP (Sigma, USA) at 1:1000 dilutions at 4°C overnight. This was followed by incubation with the goat anti-rabbit horseradish peroxidase-conjugated secondary antibody (Sigma, USA) at 1:5000 dilutions for 1 hour at room temperature. The immunoreactive proteins were visualized using enhanced chemiluminescence detection substrate (Pierce ECL western blotting substrate, Thermo Fisher Scientific, USA) in X-ray film. Expression of osteogenic proteins was quantified by densitometry (ImageJ software, National Institute of Health, Bethesda, MD). β-actin (Sigma, USA) was used as an internal control. Band density values of osteopontin, osteocalcin, and collagen type-1 were normalized with β-actin and expressions of these proteins were represented as an arbitrary ratio compared to untreated control.

For immunofluorescence, MG-63 cells were grown on collagen-coated coverslips in 6 well plates and incubated with calcium glucoheptonate for seven days. Cells were washed with PBS, fixed in 4% paraformaldehyde and permeabilized using 0.3% Triton X-100. Cells were washed, treated with blocking reagent for one hour and incubated overnight with osteopontin primary antibody in a humidified chamber at 4°C. It was further incubated with secondary antibody conjugated with Alexafluor 488 (Sigma, USA) in the dark at room temperature for one hour. Cells were washed and mounted using antifade mountant containing DAPI for counterstaining the nuclei. Images were captured using fluorescent imager (ZOE, Biorad).

### qRT-PCR analysis

MG-63 cells were treated with calcium glucoheptonate for seven days. RNA was isolated using the RNeasy RNA isolation kit (Qiagen, USA). Reverse transcription was performed to convert RNA to cDNA by using Prime Script 1^st^ strand cDNA Synthesis kit (TAKARA BIOINC, Japan). Subsequently, after reverse transcription, qPCR was performed by using cDNA, primers for Collagen type I (Forward primer: 5’ GAAGACATCCCACCAATCACC3’, Reverse primer: 5’TCTCGTCACAGATCACGTCATC3’) Osteocalcin (Forward primer: 5’TCACACTCCTCGCCCTATTG3’, Reverse primer: 5’TCGCTGCCCTCCTGCTTG3’) and SPARC (Forward primer: 5’GAAGCCCTGCCTGATGAGACA 3’, Reverse primer: 5’CCACCTCCTCTTCGGTTTCCTC 3’) genes and SYBR Green master mix in a real-time PCR machine (CFX96, BioRad, Singapore).

### Statistical analysis

Data are represented as mean ± standard deviation. For statistical comparisons, the data were analyzed by one-way analysis of variance (ANOVA) using SPSS (Version 22.0). p < 0.05 was considered statistically significant.

## Results

### Physicochemical characteristics of calcium glucoheptonate

**Fig 1:**
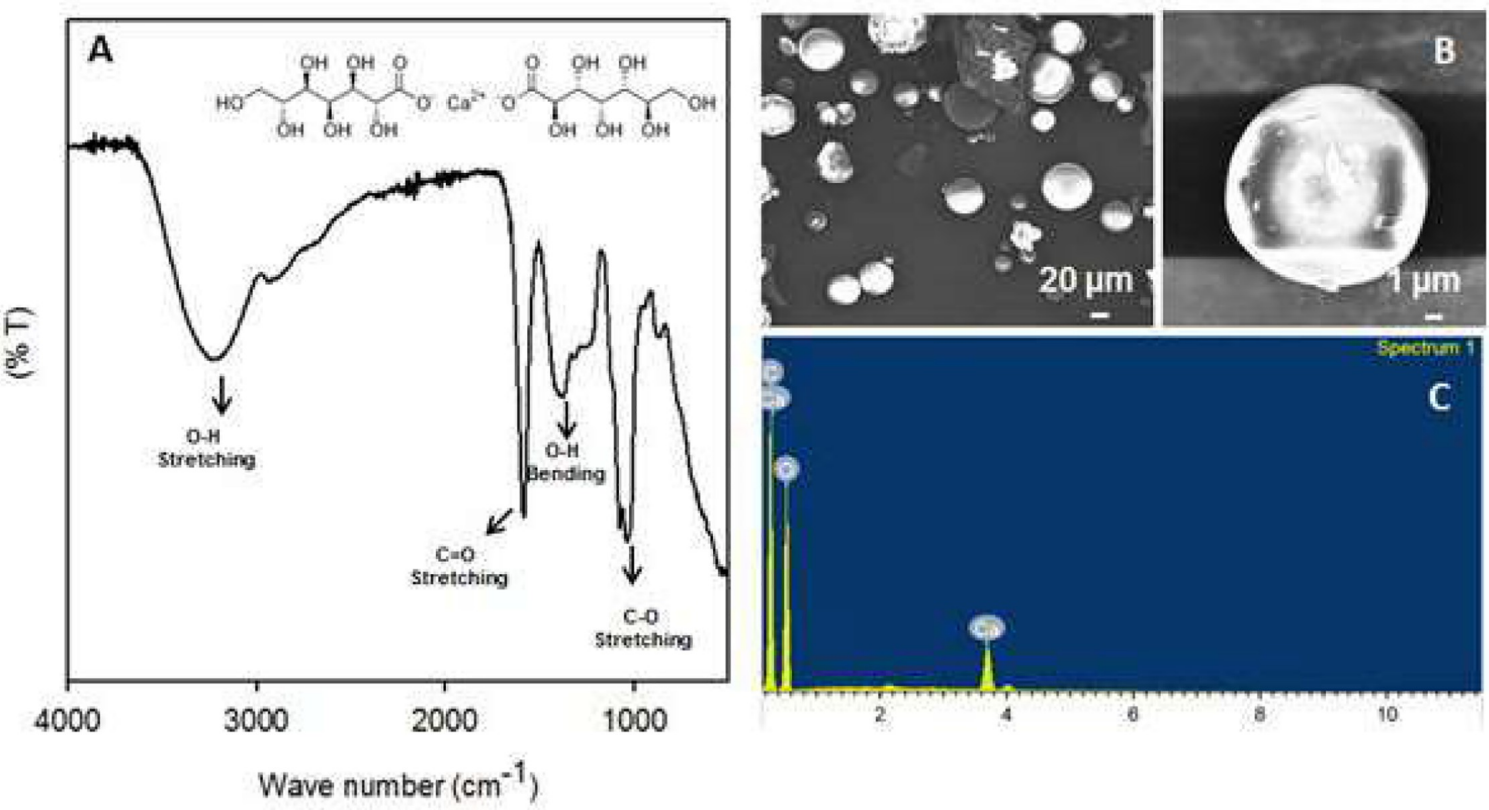
**(A)** The FT-IR spectra, (B) FESEM images and (C) EDX spectra of calcium glucoheptonate.

The chemical structure, morphology, and elemental composition of the calcium glucoheptonate were characterized by FT-IR spectroscopy and SEM- EDX analysis. Fig 1A shows all characteristic stretching and bending bands associated with different bonds. The appearance of a strong O-H broad band at 3200 cm^-1^ confirms the existence of the intermolecular bonded hydroxyl groups in the molecule. The medium O-H bending is seen at 1420 cm^-1^ indicating the hydroxyl groups. The strong stretching bands at 1710 cm^-1^ and 1085 cm^-1^ confirms the presence of C = O and C-O bonds respectively.

The surface morphological characterization by FESEM (Fig 1B) displayed uniform and micro-sized spherical morphology of the calcium glucoheptonate. The EDX analysis (Fig 1C) confirmed the presence of calcium and other expected elements. Importantly, the absence of any other elements confirmed the purity of the molecule.

### Proliferative effect of calcium glucoheptonate on osteoblast-like MG-63 cells

**Fig 2:**
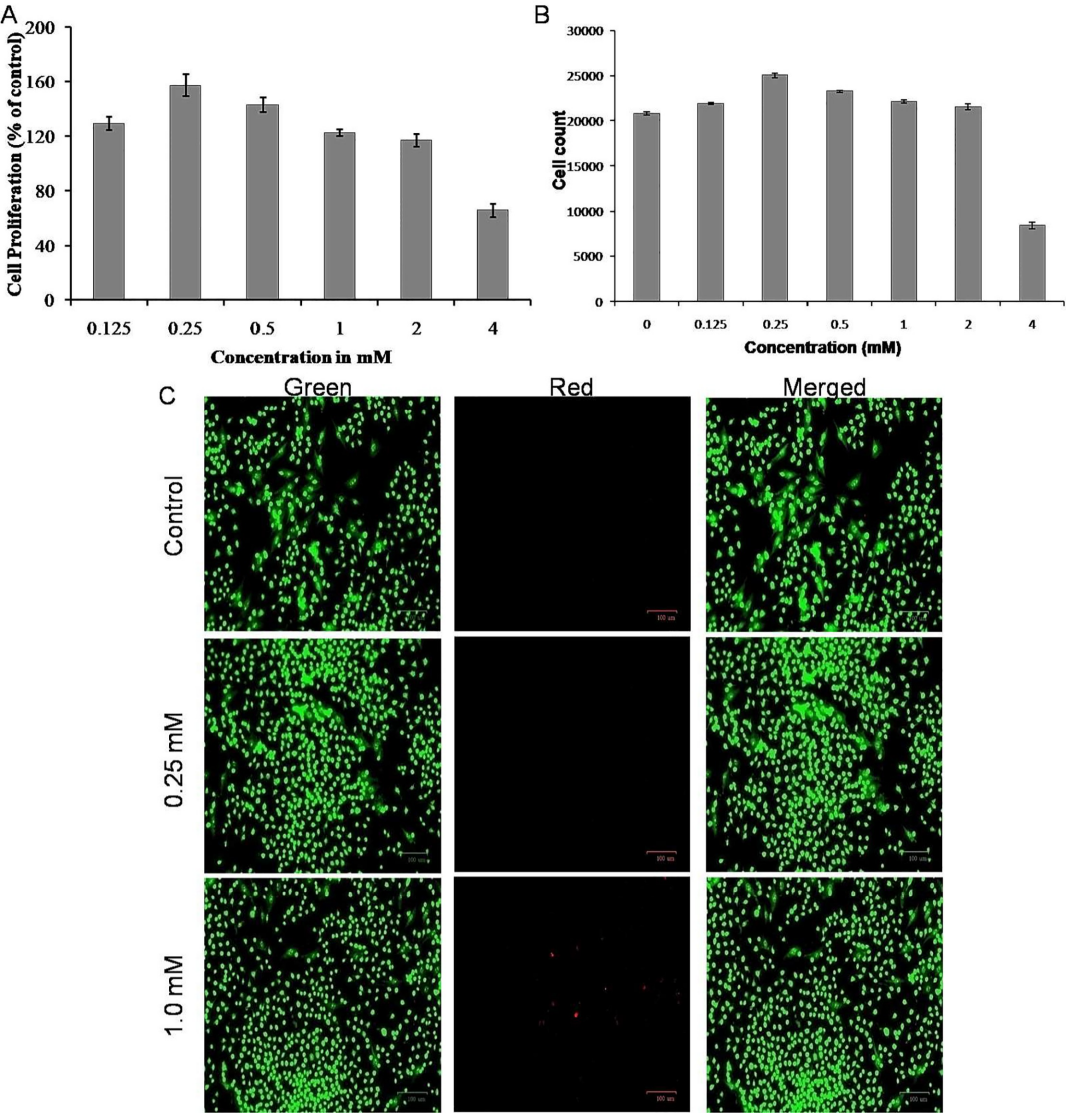
Effect of calcium glucoheptonate on the proliferation of osteoblast-like MG-63 cells. (A) MTT assay. (B) Cell counts from trypan blue dye exclusion assay. Values are mean ± SD (n = 3). (C) Photomicrographs of live/dead stained osteoblast-like MG-63 cells treated with selected concentrations of calcium glucoheptonate in comparison to untreated control. (Scale bar 100 μm). The green fluorescence represents the nuclei stained with acridine orange indicating viable cells and red fluorescence represent the nuclei stained with ethidium bromide.

The proliferative effect of calcium glucoheptonate was tested at concentrations between 0–4 mM using MTT assay. Results showed that the MG-63 cells treated with different concentrations (0.25–4 mM) of calcium glucoheptonate for 48 h had significantly higher proliferation compared to control (Fig 2A). The maximum proliferation was observed at 0.25 mM concentration (157.35% as compared to control) followed by 1.0 and 2.0 mM concentrations. However, at 4 mM concentration, there was a significant reduction in the cell viability compared to the control and other concentrations. Similar results were observed in the trypan blue dye exclusion method (Fig 2B).

Based on the MTT and trypan blue test results, two concentrations (0.25 and 1.0 mM) of calcium glucoheptonate were selected for further studies. The microscopic analysis of the MG-63 cells treated with calcium is shown in Fig 2C. The cells treated with 0.25 mM and 1.0 mM equivalents of calcium from calcium glucoheptonate showed significantly higher number of viable cells with negligible cytotoxicity.

### Calcium glucoheptonate promotes calcium uptake and mineralization

**Fig 3:**
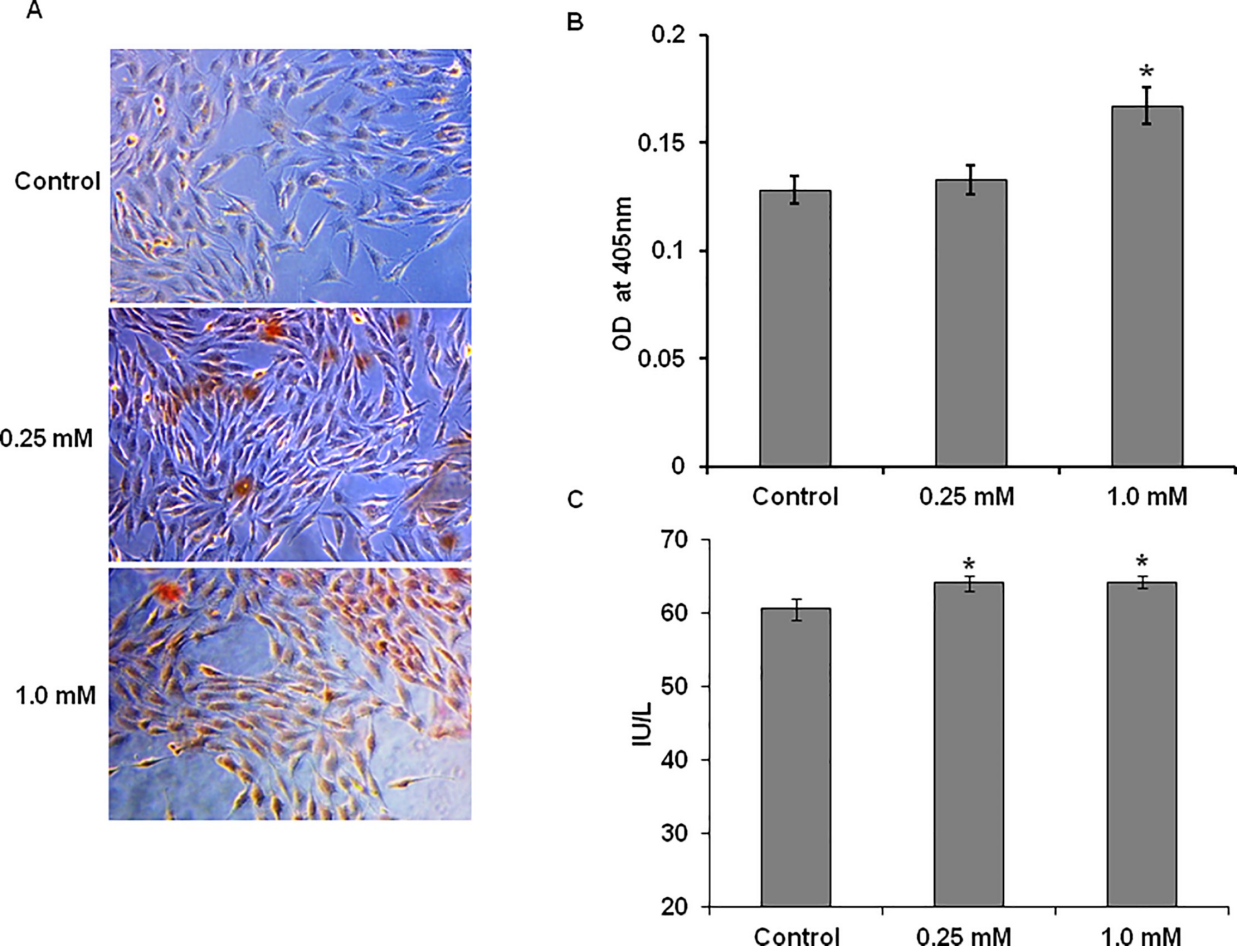
Quantification and visualization of calcium uptake and mineralization in osteoblast-like MG-63 cells. **(**A) and (B) represents Alizarin Red S staining and quantification for visualization of calcium uptake and mineralization in osteoblast-like MG-63 cells treated with selected concentrations of calcium glucoheptonate. (C) Changes in the alkaline phosphatase activity reflecting osteogenesis in osteoblast-like MG-63 cells treated with calcium glucoheptonate for seven days.

Alizarin Red S staining method was used to study the calcium accumulation in MG-63 cells treated with calcium glucoheptonate for seven days. Based on the staining intensity the calcium accumulation was quantified. The calcium accumulation by the cells increased with the dose as observed by intense red coloration due to the staining of the accumulated mineral by Alizarin Red dye (Fig 3A). The spectrophotometric quantification of Alizarin Red stain in the cells indicated higher calcium uptake and mineralization in the treatment group compared to the control. There was a 30% increase in the calcium uptake at 1.0 mM concentration compared to the control (Fig 3B). A significant increase in alkaline phosphatase activity was observed with 0.25 mM and 1.0 mM concentration of calcium glucoheptonate treatment (p < 0.05) (Fig 3C).

### Effect of calcium glucoheptonate on the expression of osteogenic genes and proteins

**Fig 4:**
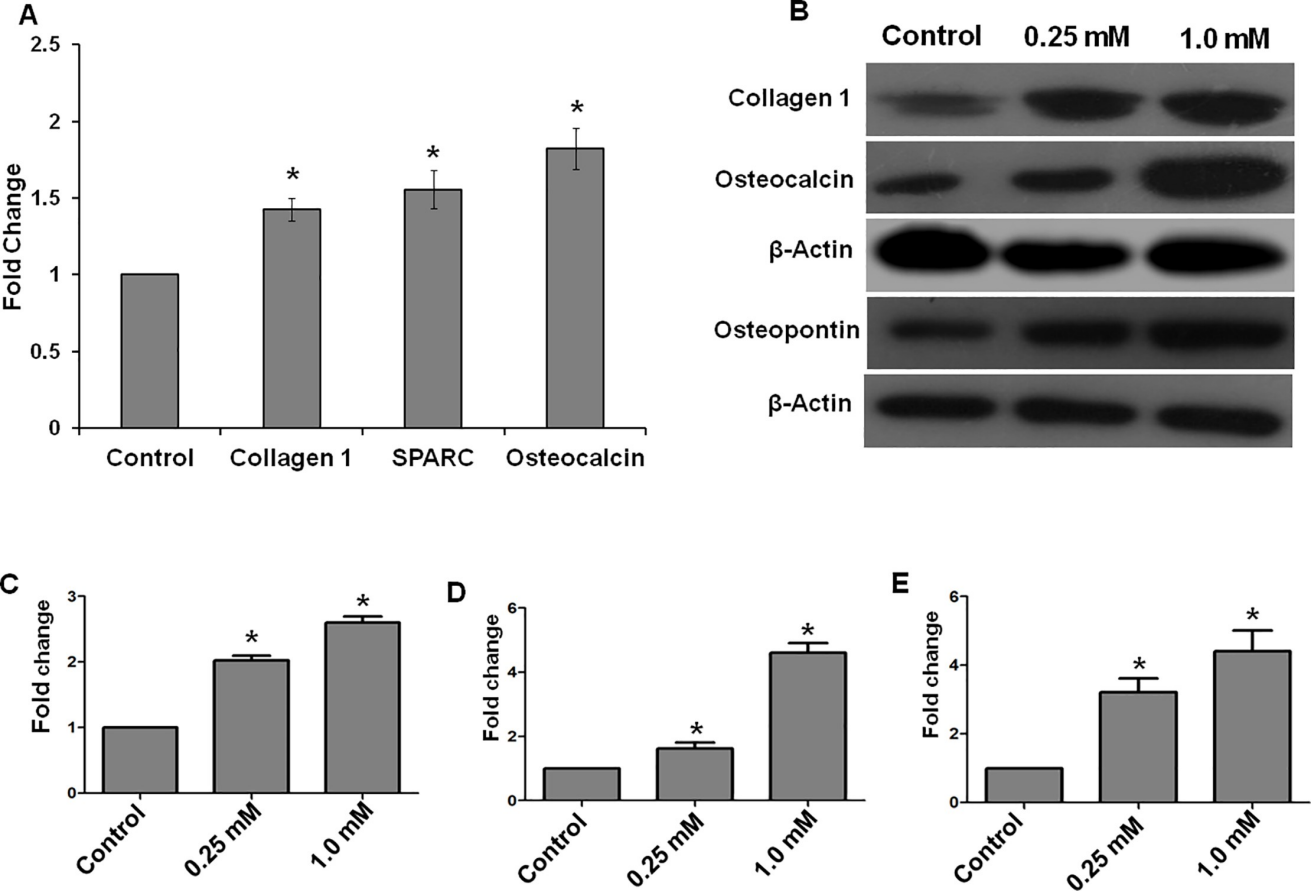
Changes in the expression pattern of osteopontin, osteocalcin, and collagen-1 in MG-63 cells treated with calcium glucoheptonate for seven days. (A) qRT-PCR results show fold-change in the expression of osteogenic genes in response to calcium glucoheptonate treatment. (B)- Western blot for collagen-1, osteocalcin and osteopontin respectively. (C-E) Densitometric analysis of collagen-1, osteocalcin and osteopontin.* denotes significant difference as compared to control (p<0.05).

The qRT-PCR experiments using collagen type I, osteocalcin and secreted protein acidic and cysteine-rich (SPARC) genes showed increased expression in the calcium-supplemented cells (Fig 4A). Treatment of MG-63 cells with 1 mM calcium glucoheptonate resulted in significant (p<0.05) upregulation of collagen type I (1.4 fold), osteocalcin (1.8 fold) and SPARC (1.5 fold) genes.

Results from the western blotting experiments showed a significant increase in osteopontin, osteocalcin and collagen-1 expression levels compared to control as shown in Fig 4B. Fig 4C–4E—show denstitomertic analysis of western blot for collagen-1, osteocalcin and osteopontin respectively. At all the tested concentrations of calcium glucoheptonate, the expression levels were higher compared to the control. Our results indicated that treatment with selected concentrations of calcium glucoheptonate enhances the osteogenic properties of MG-63 cells.

**Fig 5:**
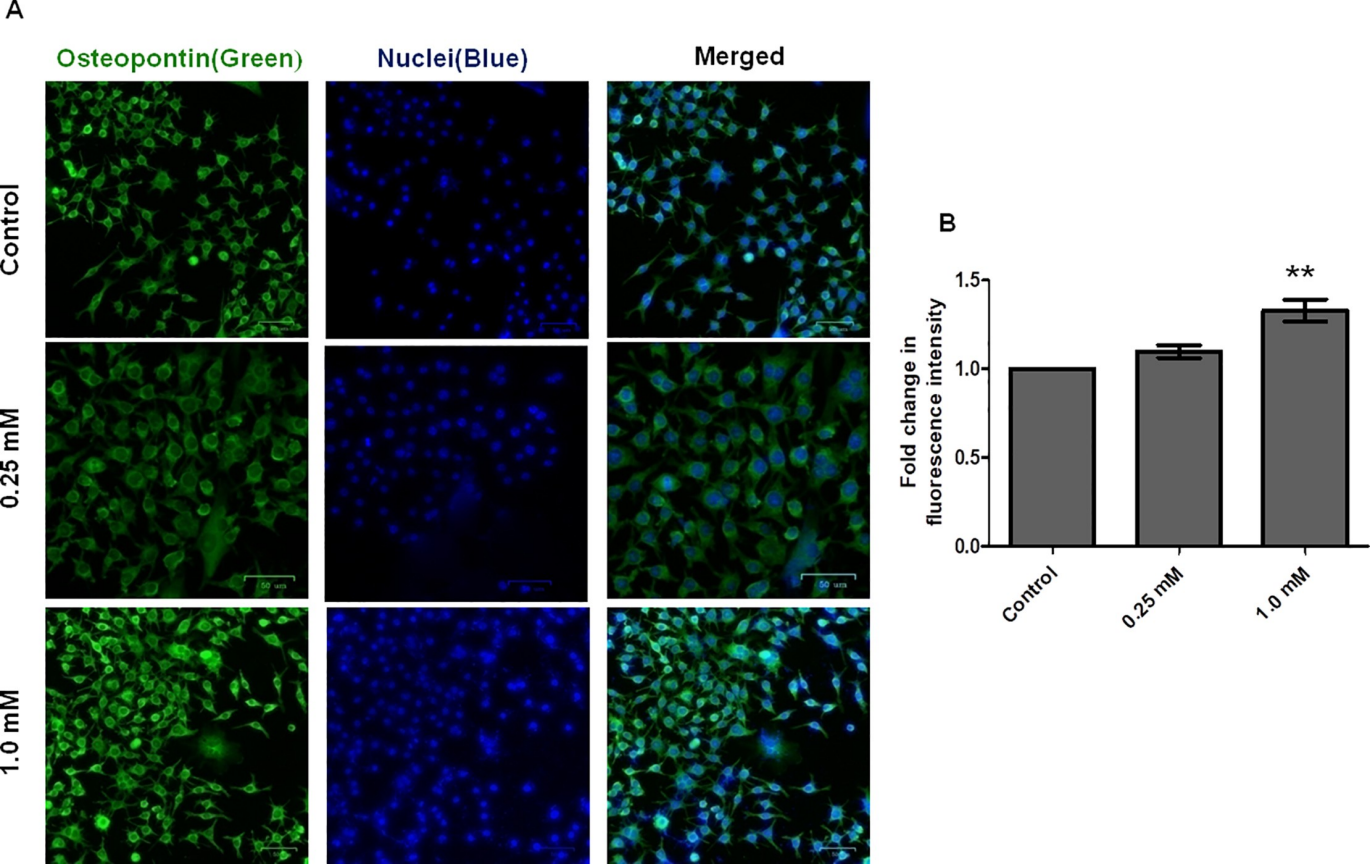
Osteopontin expression in response to calcium glucoheptonate treatment in MG-63 cells. Green fluorescence represents the expression of osteopontin and blue fluorescence indicates the nuclei counterstained with DAPI. (Scale bar: 50 μm).

Osteopontin expression in the cells was visualized using immunofluorescence assay. Cells in 0.25 mM and 1.0 mM calcium glucoheptonate treatment group showed increased levels of osteopontin expression compared to the control. Increased expression was seen at the highest tested concentration of 1.0 mM (Fig 5).

### Calcium glucoheptonate does not affect the viability of epithelial cells

**Fig 6:**
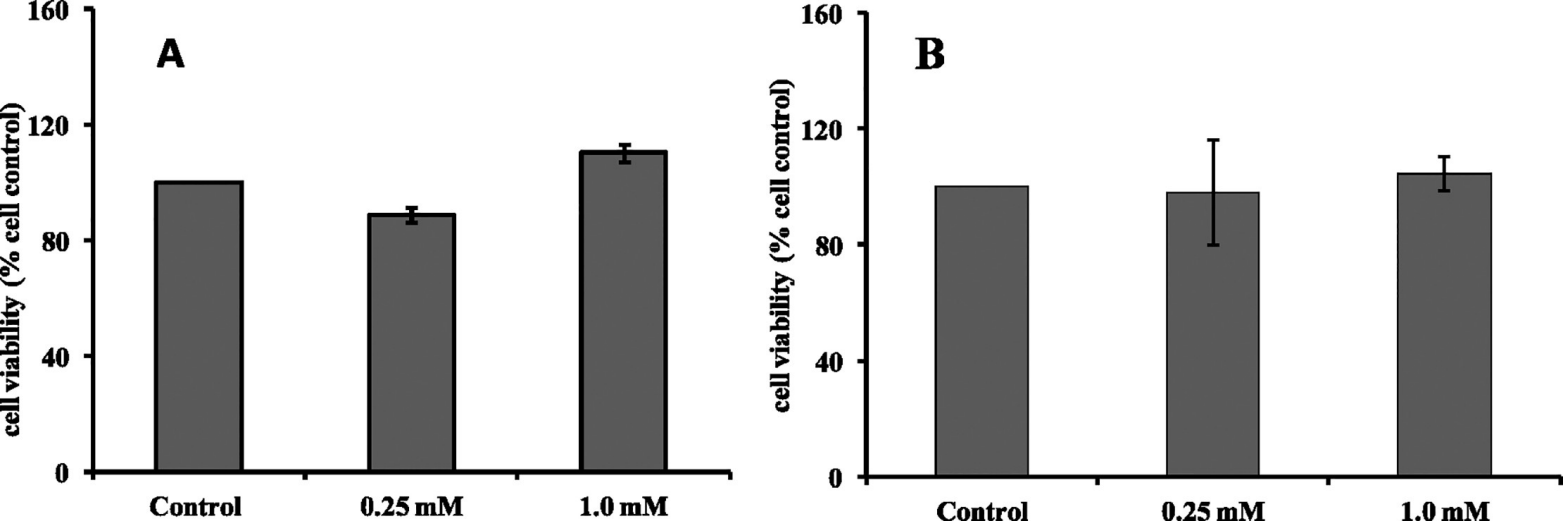
Effect of calcium glucoheptonate on epithelial cells. (A) colorectal epithelial cells (Caco2) and (B) renal epithelial cells (HEK 293T).

The epithelial cell models of Caco2 and HEK 293T were used to study the direct effect of calcium glucoheptonate. In comparison with the untreated control, Caco2 cells treated with calcium glucoheptonate showed the viability of 88.80% and 110.35% at 0.25 mM and 1.0 mM concentrations respectively (Fig 6A). Similar results were also obtained with kidney epithelial cells (HEK 293T), wherein administration of calcium glucoheptonate did not induce any cytotoxicity. The cell viability was similar to untreated control indicating no significant adverse effect on the renal cells (Fig 6B).

## Discussion

In the present study, we investigated the effect of calcium glucoheptonate on viability and proliferation of MG-63 osteoblast-like cells. MG-63 cells are osteosarcoma cells, and have been widely used as a model of osteoblast cells for osteogenic differentiation studies. [29–33]. There are reports supporting osteogenic differentiation, stemeness and tumerigenic potential of MG-63 cells [34–38].

Calcium plays an essential role in several signal transduction pathways where it works as a second messenger, involved in the release of neurotransmitter from the neurons, muscle contraction, and fertilization [1, 39]. Calcium also acts as a co-factor for many enzymes, aiding their functioning in several physiological processes such as clotting [40, 41]. Reduction in the number and activity of osteoblasts resulting in impaired osteoblastic bone formation has been implicated in osteoporosis [42]. Therefore, promoting osteoblast proliferation by mineral supplementation helps during bone remodeling in osteoporosis [43].

From our study, it is evident that calcium glucoheptonate has a positive effect on cell proliferation of osteoblast-like MG-63 cells. The MG-63 cells treated with calcium glucoheptonate for seven days showed calcium deposition, which can play a role in increase in bone mineral density [44]. Elevated calcium concentrations at the resorptive sites of bone can also regulate osteoblast functions [45]. Accumulation of calcium in the extracellular space and its organization into hydroxyapatite crystals is a key marker of osteoblast maturation [46].

Alkaline phosphatase is expressed during early development on the cell surface and in matrix vesicles. During the later developmental stages, while other genes such as osteocalcin are up-regulated, the expression of alkaline phosphatase declines [47]. Our results indicated that treatment with 0.25 and 1.0 mM calcium glucoheptonate significantly increased the alkaline phosphatase activity compared to control. Bone formation, metabolism, and regeneration are regulated by the genes COL-1 and osteocalcin which have a direct relationship with calcium availability. Osteocalcin is known to conjugate with hydroxyapatite and calcium is highly expressed in expanding skeletal tissue helping in bone mineralization and bone turnover processes [48, 49]. We observed that calcium glucoheptonate treatment up-regulated the expression of osteocalcin, COL-1, and SPARC genes. These results are in close agreement with a few previous studies, wherein, an increase in osteocalcin mRNA expression is observed due to elevated concentrations of extracellular calcium [50, 51]. Coral sand which is a rich calcium source increased COL-1 mRNA expression in Wistar rats [52].

Similarly, collagen is implicated in matrix mineralization during bone cell differentiation [53]. We observed an increase in the levels of intracellular COL-1 with calcium glucoheptonate treatment in MG-63 osteoblast-like cells, compared to control. Enhancement of COL-1 may promote the maturation and mineralization of osteoblast matrix. Osteopontin is an acidic phosphoprotein containing phosphorylated serine residues that binds to calcium crystals to modulate bone mineralization and osteogenesis [54]. It is also considered as an important factor in bone remodeling. Its function in recruiting osteoclasts to the mineral matrix of bones is imperative [55]. Results of immunofluorescence and western blotting experiments indicated the increased expression of osteopontin in calcium glucoheptonate treated cells confirming its osteogenic potential. Osteopontin binds to cell surface receptors and extracellular matrix proteins such as fibronectin, and collagen, mediating cell adhesion and migration [56]. Binding of osteopontin to calcium occurs through Runx2, 1, 25-dihydroxyvitamin D3 and Notch signaling pathways that regulate osteopontin concentration in osteoblasts and subsequent bone remodeling [57]. Calcium glucoheptonate did not have any apoptotic effect on MG-63 cells (S1 Fig).

Calcium salts may damage the epithelial cells on direct contact during the absorption process or may burden the renal system during excretion [58, 59]. Caco-2 cell line is a widely used model for the evaluating the absorption of the ingested formulations [60]. HEK 293T cells are utilized in drug induced nephrotoxicity studies [61, 62]. To test whether calcium glucoheptonate has any such adverse effect, Caco2 and HEK-293T cells were used and the results did not show any cytotoxic effect.

The overall results from the *in vitro* experiments suggest that calcium glucoheptonate can be exploited as a safe source of calcium in the pharmaceutical industry.

## Conclusion

Calcium glucoheptonate can exert cell proliferative effect on osteoblast-like MG-63 cells. The calcium released from glucoheptonate enhances the expression of collagen-1, osteocalcin and osteopontin, thereby promoting bone mineralization and osteogenesis. Calcium glucoheptonate can be a promising calcium supplement for calcium deficiency indications or for population with limited dietary calcium intake.

## Supporting information

S1 Table Details of the antibodies and the reagents used in the study. (DOC)

S1 Fig Effect of calcium glucoheptonate on apoptotic proteins. MG-63 cells were treated with 0.25 and 1.0 mM concentrations of calcium glucoheptonate. Results indicated that no apoptosis was observed upon treatment. Hydrogen peroxide treated cells were used as positive control.
(TIF)
