# Supplementary material for: Effect of calcium glucoheptonate on proliferation and osteogenesis of osteoblast-like cells in vitro
Source: PLoS One. 2019 Sep 9;14(9):e0222240. doi: 10.1371/journal.pone.0222240 (PMC6733474; doi:10.1371/journal.pone.0222240)
Supplement: S1 Table — (DOC) [file pone.0222240.s001.doc]

| Sl. No | Reagent | Manufacturer | Catalogue number | Experimental conditions |
| --- | --- | --- | --- | --- |
| 1 | Alkaline phosphatise quantification kit | Agappe Diagnostics Ltd. India) | 11401003 | As per manufacturer ‘s specifications |
| 2 | Anti osteopontin antibody | Sigma-Aldrich | O7264 | 1:1000 dilution |
| 3 | Anti osteocalcin antibody | Merck-Millipore | AB10911 | 1:1000 dilution |
| 4 | Anti collagen1 antibody | Merck-Millipore | AB745 | 1:1000 dilution |
| 5 | Anti cleaved caspase 3 antibody | Cell Signaling Technology, USA | 9664T | 1:1000 dilution |
| 6 | Anti cleaved cleaved PARP antibody | Cell Signaling Technology, USA | 5625T | 1:1000 Dilution |
| 7 | Anti β-Actin antibody | Sigma-Aldrich | A3854 | 1:50000 |
| 8 | Goat anti-rabbit horseradish peroxidase-conjugated secondary antibody | Sigma-Aldrich | A0545 | 1:5000 |
| 9 | Goat anti-rabbit secondary antibody tagged with Alexafluor 488 | Sigma-Aldrich | SAB4800234 | 1:500 |
| 10 | PrimeScript 1^st^ strand cDNA Synthesis Kit | Takara Bio USA, Inc | 6110A | As per manufacturer ‘s instruction |
| 11 | TB Green® Premix Ex Taq™ (Tli RNase H Plus) | Takara Bio USA, Inc | RR420A | As per manufacturer ‘s instruction |
| 12 | Primers for Collagen type I | Sigma-Aldrich | - | Sample volume: 25 μl  Step 1: 95℃ 30 sec  Step 2: PCR  GOTO: 39 (40 cycles)  95℃ 5 sec  60℃ 30 sec  Step 3: Melt Curve |
| 13 | Primers for Osteocalcin |
| 14 | Primers for SPARC |
| 15 | Primers for GAPDH |
