# Supplementary figures and images for: Effect of calcium glucoheptonate on proliferation and osteogenesis of osteoblast-like cells in vitro
Source: PLoS One. 2019 Sep 9;14(9):e0222240. doi: 10.1371/journal.pone.0222240 (PMC6733474; doi:10.1371/journal.pone.0222240)

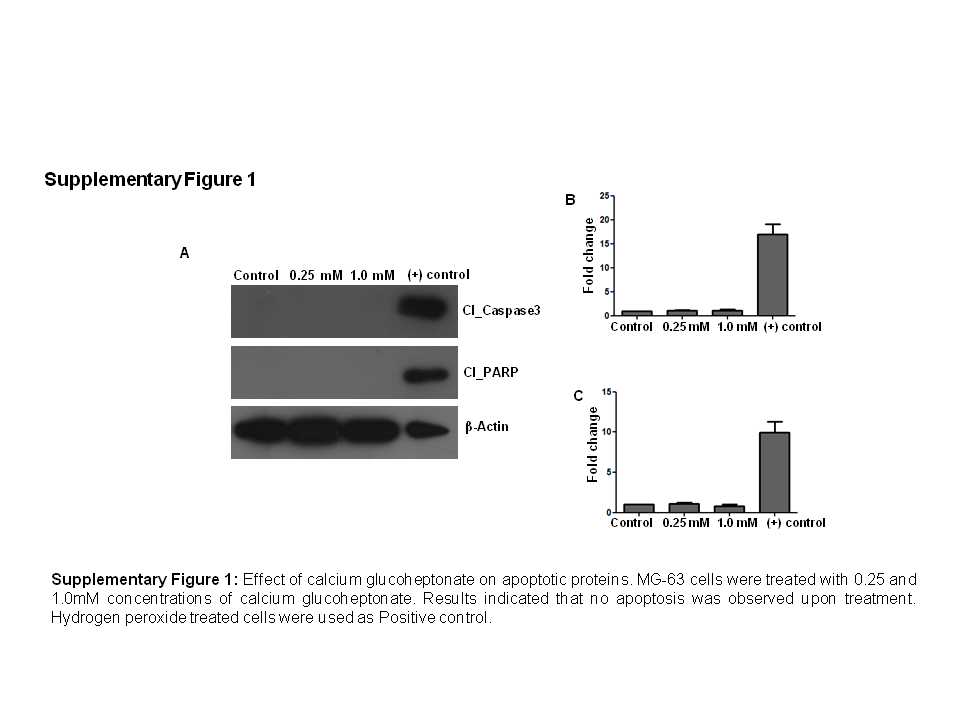

Supplement: S1 Fig — MG-63 cells were treated with 0.25 and 1.0 mM concentrations of calcium glucoheptonate. Results indicated that no apoptosis was observed upon treatment. Hydrogen peroxide treated cells were used as positive control. (TIF) [file pone.0222240.s002.tif]
